# Neddylation Regulates Class IIa and III Histone Deacetylases to Mediate Myoblast Differentiation

**DOI:** 10.3390/ijms22179509

**Published:** 2021-09-01

**Authors:** Hongyi Zhou, Huabo Su, Weiqin Chen

**Affiliations:** 1 Department of Physiology, Medical College of Georgia at Augusta University, Augusta, GA 30912, USA; wechen@augusta.edu; 2 Vascular Biology Center, Medical College of Georgia at Augusta University, Augusta, GA 30912, USA; hsu@augusta.edu

**Keywords:** neddylation, histone deacetylases, myoblast differentiation

## Abstract

As the largest tissue in the body, skeletal muscle has multiple functions in movement and energy metabolism. Skeletal myogenesis is controlled by a transcriptional cascade including a set of muscle regulatory factors (MRFs) that includes Myogenic Differentiation 1 (MYOD1), Myocyte Enhancer Factor 2 (MEF2), and Myogenin (MYOG), which direct the fusion of myogenic myoblasts into multinucleated myotubes. Neddylation is a posttranslational modification that covalently conjugates ubiquitin-like NEDD8 (neural precursor cell expressed, developmentally downregulated 8) to protein targets. Inhibition of neddylation impairs muscle differentiation; however, the underlying molecular mechanisms remain less explored. Here, we report that neddylation is temporally regulated during myoblast differentiation. Inhibition of neddylation through pharmacological blockade using MLN4924 (Pevonedistat) or genetic deletion of NEDD8 Activating Enzyme E1 Subunit 1 (NAE1), a subunit of the E1 neddylation-activating enzyme, blocks terminal myoblast differentiation partially through repressing MYOG expression. Mechanistically, we found that neddylation deficiency enhances the mRNA and protein expressions of class IIa histone deacetylases 4 and 5 (HDAC4 and 5) and prevents the downregulation and nuclear export of class III HDAC (NAD-Dependent Protein Deacetylase Sirtuin-1, SIRT1), all of which have been shown to repress MYOD1-mediated MYOG transcriptional activation. Together, our findings for the first time identify the crucial role of neddylation in mediating class IIa and III HDAC co-repressors to control myogenic program and provide new insights into the mechanisms of muscle disease and regeneration.

## 1. Introduction

Protein neddylation is a posttranslational modification in which the ubiquitin-like molecule NEDD8 (neural precursor cell expressed, developmentally downregulated 8) is covalently linked to specific lysine residues of target proteins. Neddylation requires NEDD8-specific E1, E2, and E3 enzymes [1,2,3]. The only and specific E1 enzyme for NEDD8 is a heterodimer of NEDD8 Activating Enzyme E1 Subunit 1 (NAE1 also known as APPBP1) and Ubiquitin Like Modifier Activating Enzyme 3 (UBA3). Ubiquitin Conjugating Enzyme E2 M (UBE2M, also known as UBC12) and Ubiquitin Conjugating Enzyme E2 F (UBE2F) are the E2-conjugating enzymes, where yet to be fully defined E3-ligases promote the conjugation of NEDD8 to its substrate proteins via its C-terminal glycine [2,3]. Inversely, NEDD8-specific proteases such as the COP9 signalosome (CSN) and NEDD8-Specific Protease 1 (NEDP1 also known as SENP8) remove NEDD8 from target proteins, which control the balance of the neddylation status [4,5]. Neddylation regulates a broad range of substrates that are involved in diverse cellular processes, including ubiquitin–proteasome system (UPS)-mediated protein degradation, cell cycle progression, ribosome biogenesis, transcriptional regulation and autophagy, among others [2,3]. The functions of NEDD8 in protein homeostasis have been elucidated mainly through its regulation of Cullin-RING ubiquitin ligases (CRLs), which mediate the proteolysis of ∼20% of cellular proteins [6]. Recently, neddylation has been implicated in many pathophysiological statuses such as embryonic development [7], adipogenesis [8], cardiac homeostasis [9,10], synapse formation and development [11,12], and tumor development [6]. MLN4924 (Pevonedistat; hereafter, MLN), a specific inhibitor of NAE1, shows promise in treating multiple malignances [13,14]. However, MLN has been shown to impair muscle differentiation [15]. The underlying mechanisms are not fully elucidated.

Muscle differentiation is a highly controlled, multistep process including withdrawal from the cell cycle, transcriptional activation of myotube-specific genes, and formation of multinucleated myotubes [16]. It is orchestrated by the sequential activation of four basic helix-loop-helix (bHLH) muscle regulatory factors (MRFs): Myogenic Factor 5 (MYF5), Myogenic Differentiation 1 (MYOD1), Myogenin (MYOG), and Myogenic Factor 6 (MYF6) [17] and their associations with the myocyte enhancer factor-2 (MEF2) family [18]. MYOD1 and MYF5 are initiators of the myogenic program, which upregulate the expression of MYOG and MYF6 to specify myoblasts for terminal differentiation [19]. MYOG and MYF6 then more directly trigger the expression of myotube-specific genes for myoblast fusion, the ultimate step of muscle differentiation [19].

Histone acetyltransferases (HATs) and deacetyltransferases (HDACs) act as transcriptional co-activators and co-repressors of MRFs, respectively, to control the myogenic gene transcription [20]. HDACs can be classified into four groups [21]. The class I HDACs consist of HDAC1, 2, 3 and 8. Class II HDACs contain the IIa HDAC family (HDAC4, 5, 7, and 9) and the IIb family (HDAC6 and 10). Sirtuins (SIRT1, 2, 3, 4, 5, 6, and 7), a family of NAD+-dependent deacetylases, form the class III HDACs. HDAC11 is the sole class IV HDAC [21]. HDAC1 directly binds and inhibits MYOD1 activity in undifferentiated myoblasts [22]. Upon differentiation, HDAC1 expression is downregulated, while the formation of the pRb–HDAC1 complex induces the disruption of the MYOD1–HDAC1 complex, then the transcriptional activation of muscle-specific genes [23]. In proliferative myoblasts, class II HDACs, such as HDAC4, 5, and 7, associate with and inhibit the activity of MEF2 family members, thereby blocking MYOD1-dependent conversion of myoblasts into myotubes [16,24]. Likewise, SIRT1 directly or indirectly regulates MYOD1 activity through its association with a MYOD1/PCAF/GCN5 complex to inhibit myogenesis [25,26]. SIRT1 expression is downregulated and also shuttles from the nucleus to the cytoplasm during muscle differentiation [27]. Various post-translational modifications (PTMs) regulate HDAC levels and functions, including acetylation, methylation, phosphorylation, sumoylation, and ubiquitination [21]. However, the potential involvement of neddylation in regulating HDACs thus muscle differentiation has not been described.

In this study, we, for the first time, showed that neddylation inhibition blocks terminal myogenic differentiation of C2C12 cells through enhancing HDAC4/5 and SIRT1 expression and/or preventing SIRT1 nuclear export to inhibit MYOG and muscle-specific gene expression.

## 2. Results

### 2.1. Neddylation Is Downregulated during Myoblast Differentiation

**Figure 1:**
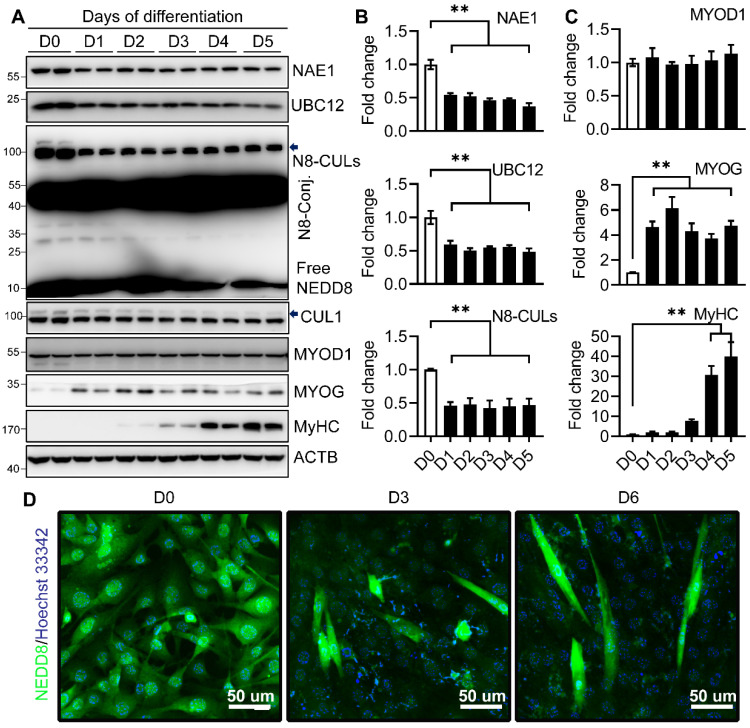
Neddylation is differentially regulated during C2C12 differentiation. (**A–C**) Western blot of the indicated proteins (**A**), and quantifications of NAE1, UBC12, and neddylated Cullins (N8-CULs) (**B**); quantifications of MYOD1, MYOGENIN, and MYOSIN (skeletal, fast) (MyHC) (**C**) in proliferative C2C12 cells cultured in growth media (i.e., day 0, D0) and for the indicated days after differentiation induction (D1 to D5). Proteins were quantified and normalized to ACTB. Data were presented as fold changes with D0 cells set at 1. N8-Conj.: NEDD8-conjugated. Arrows indicate neddylated CULs and CUL1. (**D**) Representative immunofluorescence images of C2C12 cells stained with NEDD8 (green) in growth media (D0) and for 3 and 6 days in differentiation media (D3, D6). Nuclei were counterstained with Hoechst 33342. Scale bars: 50 μm. ** *p* < 0.005 versus D0. One-way ANOVA. Three independent experiments.

To determine the role of neddylation in muscle differentiation, we first examined the neddylation profile changes during the terminal myoblast differentiation using C2C12 cells. We found that the protein levels of NAE1 and UBC12 were decreased by 50% upon induction of differentiation at day 1 (D1) and maintained the same low levels until myotube maturation by D5 (Figure 1A,B). The levels of total NEDD8-conjugated (N8-Conj.) proteins including neddylated Cullins (N8-CULs) as well as the free NEDD8 also showed similar downregulation during myoblast differentiation (Figure 1A,B). Interestingly, an unknown NEDD8-conjugated protein at ≈50 kD was highly abundant and appeared unaltered in C2C12 cells during the differentiation. The reduced expression of neddylation enzymes was further confirmed by the concomitant decrease of neddylated CUL1 upon induction of myogenesis (Figure 1A). As reported before, the protein expression of MYOD1 did not show changes during terminal myoblast differentiation, whereas the expression of MYOG and myosin heavy chain (MyHC), the early and late differentiation markers, were significantly upregulated in the early and late phases of differentiation, respectively (Figure 1A,C), which is indicative of the successful myotube formation. Interestingly, when we specifically stained the C2C12 with NEDD8 during myoblast differentiation, we identified that the levels of neddylated proteins were higher in proliferative C2C12 cells. Its level was downregulated in most differentiating but non-fused C2C12 cells. However, higher neddylation levels were very prominent in fused myotubes at both D3 and D6 of differentiation (Figure 1D). These results emphasize that activation of the myoblast differentiation program is coupled to the partial inhibition of neddylation, especially at the entry of myoblast differentiation. However, enhanced neddylation is associated with the successful formation of myotubes.

### 2.2. Neddylation Inhibition Blocks Myotube Formation

**Figure 2:**
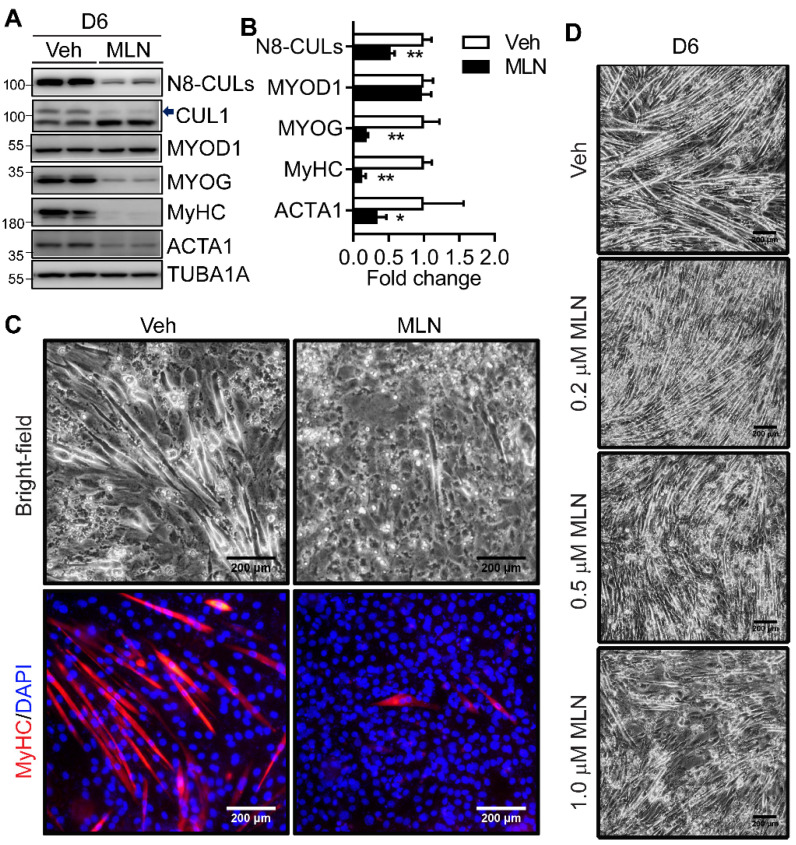
Neddylation inhibition mediated by a specific NAE1 inhibitor MLN4924 blocks C2C12 myotube formation. (**A**,**B**) Western blot analysis of neddylated Cullins (N8-CULs), CUL1, and muscle differentiation markers in vehicle (Veh)-treated and MLN4924 (MLN, 0.5 μM)-treated C2C12 for 6 days (D6) in differentiation media. Proteins were quantified and normalized to TUBA1A. Arrow indicates neddylated CUL1. Data were presented as fold changes with Veh-treated cells set at 1. * *p* < 0.05; ** *p* < 0.005 vs. Veh. Unpaired t tests. (**C**) Representative bright-field and immunofluorescence images of Veh-treated and MLN-treated C2C12 at D6 of differentiation. Cells were stained with MyHC (red), and nuclei were counterstained with DAPI (blue). Scale bar: 200 μm. (**D**) Representative bright-field images of Veh- and MLN-treated C2C12 cells at D6 in differentiation media with insulin. MLN at indicated doses was only treated for the first 2 days of differentiation. Scale bar: 200 μm. Three independent experiments.

Next, we examined whether neddylation inhibition affects myotube formation. We applied a specific NAE1 inhibitor MLN throughout the program of C2C12 differentiation. By D6 of differentiation, MLN inhibited NAE1-mediated neddylation by 50% as validated by a reduction in neddylated CULs as well as the specific CUL1 (Figure 2A). The increased expression of native CUL1 was due to the impaired CUL1 degradation when its neddylation was inhibited (Figure 2A). Interestingly, MLN treatment completely repressed the myotube formation as assessed by the dramatically diminished protein expression of MYOG, MyHC, and α-skeletal actin (ACTA1), but not MYOD1 (Figure 2A,B). Consistent with the failure to form multinucleated myotubes, bright-field microscopy as well as immunofluorescence staining of MyHC further confirmed the successful formation of multinucleated, myosin-expressing myotubes in differentiated vehicle-treated, but not MLN-treated, C2C12 cells (Figure 2C). MLN treatment for the first two days of differentiation also dose-dependently reduced myotube formation (Figure 2D), suggesting the importance of maintaining neddylation during the early stage of myoblast differentiation. These data reveal that neddylation is necessary to ensure the successful myotube formation.

### 2.3. NAE1 Deletion in C2C12 Cells Prevents Terminal Myoblast Differentiation

**Figure 3:**
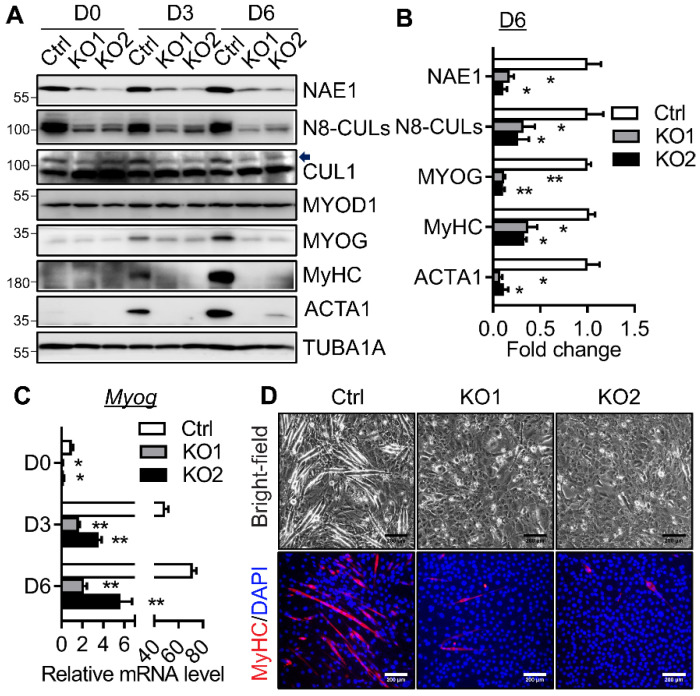
Neddylation inhibition mediated by knockout of NAE1 inhibits C2C12 differentiation. C2C12 cells were infected with lentiviruses expressing gRNAs (gRNA1 and gRNA2) against murine *Nae1*, respectively, to generate bulk cultures of cells with NAE1 knockout (KO1 and KO2). Cells infected with lentiviruses expressing no gRNA were used as controls (Ctrl). (**A**) Western blot analysis of NAE1, neddylated Cullins (N8-CULs), CUL1, myogenic transcription factors, and muscle differentiation markers during the differentiation. Arrow indicates neddylated CUL1. (**B**) Quantifications of protein expressions as normalized to TUBA1A at D6. Data were presented as fold changes with Ctrl set at 1. (**C**) qRT-PCR analysis of *Myog* gene expression at D0, D3, and D6 of myoblast differentiation. (**D**) Representative bright-field and immunofluorescence images at 6 days in differentiation medium. Cells were stained with MyHC (red), and nuclei were counterstained with DAPI (blue). Scale bar: 200 μm. * *p* < 0.05; ** *p* < 0.01 vs. Ctrl. One-way ANOVA. Three independent experiments.

To further illustrate the essential role of neddylation in myoblast differentiation, we generated NAE1-knockout (KO) C2C12 cells using the CRISPR/Cas9 strategy. Lentiviruses overexpressing two gRNAs (gRNA1 and gRNA2) targeting exon 2 and 3 of the murine *Nae1* gene, respectively, were used to infect proliferating C2C12 cells to delete NAE1 and generate two lines of NAE1-KO C2C12 myoblasts. C2C12 cells infected with lentivirus expressing no gRNA were used as a control (Ctrl). We confirmed the successful deletion of NAE1, which led to the reduced neddylated Cullins including CUL1 in NAE1-KO C2C12 cells throughout the myogenesis (Figure 3A). The protein expression of MYOD1 was not perturbed in proliferative and differentiated NAE1-KO C2C12 cells. Interestingly, NAE1 deletion slightly downregulated the mRNA levels of MYOG without altering its protein expression in D0 proliferative C2C12 cells (Figure 3A,C). Both mRNA and protein levels of MYOG were drastically upregulated at D3 and D6 in Ctrl cells (Figure 3A,C). However, such upregulations were almost completely blunted in differentiating NAE1-KO C2C12 cells as compared to Ctrl cells (Figure 3A,C; D6 protein levels were quantified in Figure 3B). Accordingly, the expressions of the late differentiation markers (e.g., MyHC and ACTA1) were significantly reduced at D3 and D6 in NAE1-KO C2C12 cells (Figure 3A, with D6 protein quantified in Figure 3B). Bright-field microscopy and immunofluorescence staining of MyHC further confirmed a nearly complete lack of myotube formation by six days of differentiation in NAE1-KO C2C12 cells (Figure 3D). These data suggest that NAE1 deletion blocks neddylation, which impairs myotube formation partially through repressing MYOG upregulation, similar to the effect of MLN.

### 2.4. Neddylation Deficiency Blocks Myoblast Differentiation Potentially through Modulating Class II and III HDACs

**Figure 4:**
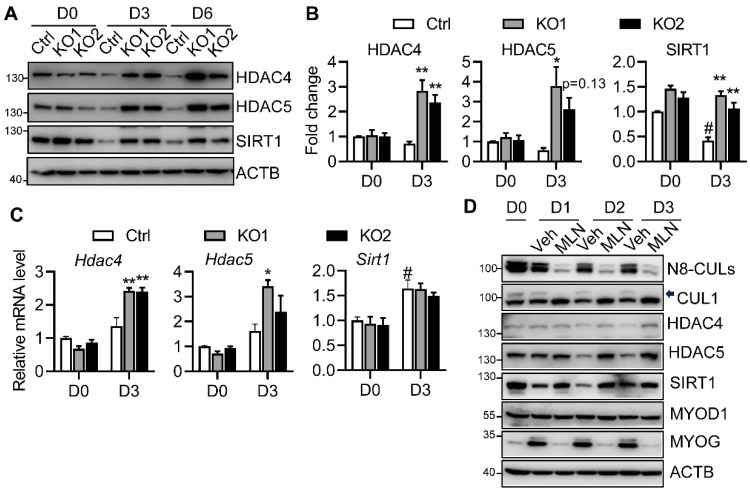
Neddylation controls the expression of class IIa and class III HDACs during myoblast differentiation. (**A**) Western blots of HDAC4, 5 and SIRT1 in NAE1-KO C2C12 cells before (D0) and after 3 and 6 days (D3 and D6) of myoblast differentiation as compared with vector controls (Ctrl). (**B**) Protein expressions at D0 and D3 were quantified by normalizing to ACTB. Data were presented as fold changes with D0 Ctrl cells set at 1. (**C**) qRT-PCR gene expression analysis of *Hdac4/5* and *Sirt1* at D0 and D3 of myoblast differentiation. D0 Ctrl cells were set at 1. * *p* < 0.05; ** *p* < 0.005 vs. Ctrl at the same day of differentiation. # *p* < 0.05 vs. D0 Ctrl cells. Two independent experiments in triplicates. (**D**) Western blots of neddylated Cullins (N8-CULs), CUL1, HDAC4, 5, and SIRT1, and myogenic transcription factors in vehicle (Veh) and 0.5 μM MLN4924 (MLN) treated C2C12 cells before and during the first three days of myogenic differentiation. Arrow indicates neddylated CUL1. Three independent experiments.

MYOG contains both MYOD1 and MEF2 binding sites at its promoter. MEF2 recruits HDAC4/5 to the MYOG promoter, which inhibits MYOD1 activity and therefore blocks MYOG expression and, thus, myogenesis [16,28]. Likewise, activation of SIRT1 also inhibits myogenesis by direct or indirect interactions with the MYOD1 and MEF2 [25,29]. We consistently observed unaltered MYOD1, but highly blunted MYOG, expression, suggesting that inhibition of terminal myoblast differentiation by neddylation deficiency at least partially occurs at the level of MYOG potentially through perturbing its upstream MYOD1 activity, not its expression. Therefore, we focused on the negative regulators of MYOD1: class II and III HDACs. In the Ctrl cells, induction of myoblast differentiation led to a tendency of lower protein expressions of HDAC4/5 by D3 (Figure 4A,B), which cannot be attributed to the changes in their mRNA levels (Figure 4C). NAE1 deletion did not perturb the protein levels of HDAC4/5 in D0 proliferative NAE1-KO C2C12 cells as compared to the Ctrl myoblasts. However, the protein expressions of HDAC4/5 at D3 of differentiation were significantly enhanced in both lines of NAE1-KO C2C12 cells (Figure 4A,B), which were largely due to their upregulated mRNA levels (Figure 4C). In contrast, SIRT1 was reduced by 60% in D3-differentiating Ctrl cells. However, NAE1 deletion prevented SIRT1 from downregulation upon myogenic induction, which occurred specifically at protein, not mRNA, levels (Figure 4A–C). We further examined the earlier responses of HDAC4/5 and SIRT1 in MLN-treated C2C12 cells during the first three days of myoblast differentiation. As expected, MLN inhibited neddylation of CULs, such as CUL1 (Figure 4D). Not surprisingly, elevated protein expressions of HDAC4/5 in MLN-treated C2C12 cells were obvious by D3 of differentiation (Figure 4D). Again, SIRT1 was rapidly downregulated within 24 h of differentiation in vehicle-treated cells; its level was sustained in MLN-treated differentiating C2C12 cells (Figure 4D). No significant changes in MYOD1 levels were observed, whereas MLN-treated cells largely failed to express MYOG at as early as D1 after induction of differentiation (Figure 4D). These data further illustrate that neddylation deficiency blocks entry of terminal myoblast differentiation at the MYOG step at least partially due to the upregulation of the well-known negative regulators of MYOG transcription: HDAC4/5 and SIRT1, which ultimately inhibits myotube formation in C2C12 cells.

### 2.5. Neddylation Deficiency Causes SIRT1 Accumulation in the Nucleus during the Early Stage of Myoblast Differentiation

**Figure 5:**
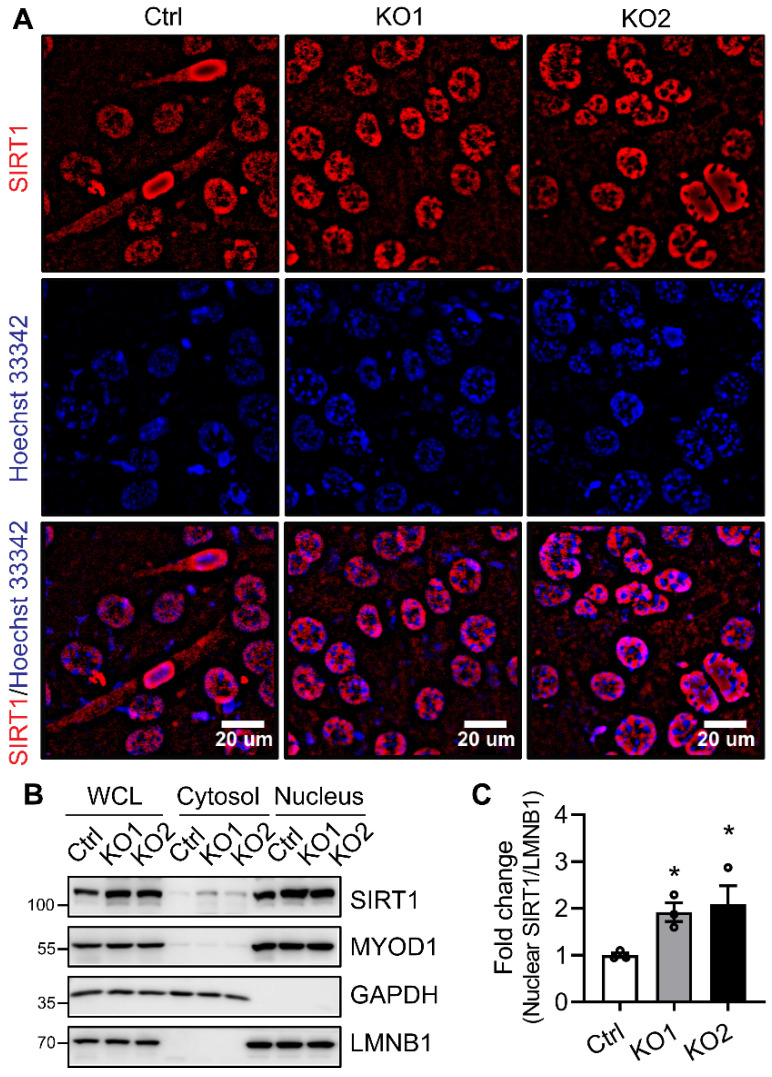
Neddylation inhibition dysregulates SIRT1 intracellular localization. (**A**) Representative immunofluorescence images of Ctrl and NAE1-KO (KO1, KO2) C2C12 cells at 3 days in differentiation media. Cells were stained with SIRT1 (red), and nuclei were labeled with Hoechst 33342 (blue). Scale bar: 20 μm. (**B**) Western blots of whole cell lysates (WCL), cytosol, and nucleus fractions of Ctrl and NAE1-KO (KO1 and KO2) C2C12 cells at 3 days in differentiation media. GAPDH and LMNB1 were used as cytosol and nucleus marker proteins, respectively. (**C**) Quantifications of nuclear fraction of SIRT1 as normalized to LMNB1 with Ctrl set as 1. * *p* < 0.05 vs. Ctrl. One-way ANOVA. Three independent experiments.

Downregulation of SIRT1 in differentiating C2C12 cells acts mainly via a post-translational manner (Figure 4A–C). Failure to downregulate SIRT1 during myoblast differentiation of both MLN-treated and NAE1-KO C2C12 cells suggests a potential involvement of neddylation in regulating SIRT1 stability. Meanwhile, the localization of SIRT1 in the nucleus is also critical for inhibiting myogenesis. Next, we focused on comparing whether the sustained SIRT1 level mainly occurred in the nucleus of NAE1-KO C2C12 cells during early myogenesis. At D3, some of the Ctrl cells have already formed differentiating myotubes, which still contained a large amount of nuclear SIRT1, although mainly in the periphery of the nucleus. More SIRT1 was localized in the cytoplasm of Ctrl D3 myotubes than the residual unfused myoblasts, suggesting that SIRT1 shuttles from the nucleus to the cytoplasm when myoblasts are triggered to differentiate (Figure 5A). In contrast, in D3 NAE1-KO differentiating C2C12 cells, whereas no myotubes were detectable, SIRT1 was mostly retained in the nucleus. In addition, the nuclear SIRT1 immunofluorescence were significantly intensified when compared to the D3 Ctrl cells (Figure 5A), consistent with the augmented SIRT1 expression in neddylation-deficient C2C12 cells during differentiation (Figure 4A). We also performed cytosol/nucleus fractionation to detect the subcellular localizations of SIRT1 during myoblast differentiation. In line with the immunofluorescence staining, SIRT1 was mainly nuclear in Ctrl C2C12 cells; the expression of SIRT1 was increased in both cytoplasmic and nuclear fractions in NAE1-KO C2C12 cells three days after switching to the differentiation media (Figure 5B,C). There were no differences in total and nuclear MYOD1 levels, further suggesting that inhibition of myocyte differentiation may occur through repressing MYOD1 activity via more nuclear SIRT1. These data suggest that neddylation inhibition increases SIRT1 nuclear localization to block the muscle differentiation.

## 3. Discussion

In this study, we found that neddylation is temporally regulated in association with myogenic differentiation of C2C12 cells. It is initially downregulated but selectively elevated in fused myotubes. Pharmacological inhibition of neddylation or genetic deletion of NAE1, the subunit of only E1 enzymes, led to complete failure of myotube formation. Mechanistically, neddylation deficiency resulted in an early upregulation of myogenic co-repressors such as HDAC4/5 and SIRT1 as well as SIRT1 nuclear retention to inhibit MYOG expression and thus myogenesis. These studies provide insights into an essential role of an understudied post-translational modification, neddylation, in mediating myoblast differentiation and thus muscle cell mass and regeneration.

A previous study demonstrated that neddylation is transiently upregulated during the first two days of myoblast differentiation and that it returned to the similar levels as the proliferative myoblasts [15]. In contrast, we performed more comprehensive Western blot studies of the whole cell lysates, which identified reduced levels of neddylated proteins concomitant with lower expressions of the enzymatic machineries of neddylation, including NAE1 (E1), UBC12 (E2), and free NEDD8, throughout the myoblast differentiation. It remains unknown whether such discrepancies are caused by different cell culture conditions or by the antibodies used. Interestingly, when performing immunofluorescence staining, which provides a more cell-specific neddylation profile, in more mixed populations of fused or non-fused differentiating myoblasts, we, for the first time, identified a strikingly higher level of NEDD8 staining in fused myotubes, but markedly reduced neddylation levels in nonfused differentiating myoblasts. Thus, the overall reduction in neddylation status in D5-6 differentiated C2C12 cultures may be due to the lower efficiency of myotube formation induced by standard myoblast differentiation protocol. Nevertheless, our study highlights an early downregulation of neddylation upon initiation of myoblast differentiation, yet an enhanced neddylation in mature myotubes, suggesting neddylation is tightly involved during different stages of myoblast differentiation.

Inhibition of neddylation by MLN or siRNA-mediated NEDD8 knockdown prevents myotube formation in C2C12 cells [15]. Our study first recapitulated the potent effect of MLN in blocking myoblast differentiation. We further utilized CRISPR/Cas9 strategies to delete NAE1 in C2C12 cells, which consequently inhibited neddylation and completely shut down the terminal myoblast differentiation program (Figure 3). These data clearly emphasize the essential role of neddylation in regulating myotube formation. Furthermore, we and others also illustrated the importance of neddylation for the onset of myoblast differentiation, as inhibiting neddylation during the first two days of myogenesis effectively blocked myotube formation ([15] and Figure 2D). While the physiological role of downregulating neddylation upon myoblast differentiation remains unknown, it seems necessary to maintain certain levels of neddylation to ensure the successful myogenic program.

A previous study attributes the blockage of myoblast differentiation caused by neddylation deficiency largely to the dysregulated CRL activities [15]. However, thus far, no specific CRL substrates have been identified to account for the observed phenotype. MYOD1 and MYF5 are two critical bHLH factors that have overlapping functions in specification of myoblasts; deletion of both genes completely eliminates the skeletal muscle formation [30]. In our study, we found neddylation deficiency did not perturb the mRNA and protein expressions of MYOD1 (Figure 2 and Figure 3) and MYF5 (not shown). MYOG acts downstream of MYOD1 and MYF5 to switch on muscle differentiation genes, the absence of which resulted in a severe deficiency of muscle fibers despite similar muscle cell commitment [31,32]. MYOG itself is degraded by UPS involving SCF (Skp1-Cullin1-F-box protein) ubiquitin ligase [33]. However, inhibition of neddylation caused a complete block of MYOG at both mRNA and protein levels (Figure 2 and Figure 3). These data suggest that neddylation deficiency did not perturb MYOG protein stability via targeting CRLs; rather, it may act at least partially through repressing MYOD1 transcriptional activity to downregulate MYOG transcription and expression. Therefore, we reason that neddylation may be required for the degradation of co-repressors of MYOD1 to regulate myogenic transcription.

HDACs are well-known co-repressors directly or indirectly involved in suppressing MYOD1 transcriptional activities. After induction of differentiation, HDACs leave the promoters of muscle-specific genes through decreased expression, nuclear export, and/or a redistribution to other binding proteins to facilitate MYOD1/MEF2-mediated muscle transcription activation [34,35,36]. Several HDACs, including HDAC1 [37,38], HDAC2 [39], HDAC4/5 [40], HDAC6 [41], and SIRT1 [42,43], have been shown to be either regulated by the proteasome pathway or neddylation itself. In our study, qRT-PCR and Western blotting screening of various HDACs in MLN-treated or NAE1-KO C2C12 cells identified that only class IIa HDAC4/5 and SIRT1, and not the other HDAC1, 3, 6, and 9, were accumulated at the onset of myoblast differentiation (Figure 4 and data not shown). As previously reported, HDAC4/5 [34] and SIRT1 [27] protein levels decrease in differentiating myoblasts. However, both HDAC4 and HDAC5 are increased on the transcriptional and on the protein levels in differentiating neddylation-deficient C2C12 cells (Figure 4). Different from the mRNA upregulation of *Hdac4/5*, the sustained higher levels of SIRT1 in NAE1-KO and MLN-treated differentiating C2C12 cells seem to be mainly at the posttranscriptional level, potentially due to the failure to block the active degradation of SIRT1. Meanwhile, SIRT1 is reported to translocate from the nucleus to the cytoplasm after the induction of muscle differentiation [27]. Such export was also dysregulated when neddylation is inhibited, although it is hard to determine whether more nuclear accumulation of SIRT1 is secondary to the overall higher SIRT1 levels. This requires further studies to dissect whether neddylation deficiency alters phosphatidylinositol-3-kinase (PI3K)-mediated SIRT1 phosphorylation, which regulates SIRT1 nuclear export [27]. In addition, we used class II HDAC inhibitors (LMK-235 and MC1568) and SIRT1 inhibitors (EX-527 and nicotinamide), which failed to rescue differentiation in neddylation-inhibited C2C12 cells (data not shown). Such results are not surprising considering the broad substrates of neddylation, which renders it almost impossible to target one single pathway to revert the phenotype. Notably, all these changes occurred after the initiation of differentiation, further emphasizing the importance of neddylation in ensuring the proper myogenic program. Thus far, HDAC4/5 [40] and SIRT1 [42,43] have all been shown to be regulated by ubiqutination-mediated proteasome pathways. Exactly how neddylation, in combination with the differentiation signal, directly or indirectly regulates these myogenic co-repressors awaits further investigation.

In conclusion, our study strengthens the importance of neddylation in controlling proper myogenic program and uncovers a new mechanistic link between neddylation and the myogenic co-repressors HDACs. Our study also cautions that clinically utilizing MLN4924 to inhibit neddylation for cancer treatment may cause unexpected effects on muscle homeostasis in patients.

## 4. Materials and Methods

### 4.1. Cell Culture and Myoblast Differentiation and Reagents

C2C12 cells (ATCC# CRL-1772) were maintained in Dulbecco’s modified Eagle’s medium (DMEM) containing 10% fetal bovine serum (Hyclone, Thermo Fisher Scientific, Waltham MA, USA) and 1% penicillin-streptomycin (DMEM growth medium) at 37 °C incubator with 5% CO_2_. Differentiation was induced in DMEM plus 2% horse serum (Hyclone) and 1% penicillin-streptomycin (DMEM differentiation medium) after cells reached confluence. The differentiation medium was changed every day. MLN4924 compound was purchased from Millipore Sigma and dissolved in DMSO for storage at −20 °C.

### 4.2. CRISPR/Cas9 Gene Targeting, Lentivirus Production, and Infection of C2C12 Cells

For lentivirus production, 293T cells were transfected with the guide RNA (gRNA) plasmid (pLenti-CRISPR V2 empty vector or vector-encoding gRNA1 and gRNA2 against murine Nae1, GenScript), pMD2.G-VSV-G, and psPAX2 using PolyJetTM in vitro DNA transfection reagent (Signagen Laboratories). The two gRNA sequences are: gRNA1, TATAGGCTGTGGGGTGATCA and gRNA2, GAACCGAGCTCAAGCTGCCA. Two days later, lentivirus-containing supernatants were filtered through a 0.45 µm filter and then combined with fresh DMEM complete medium at a 1:1 ratio to infect proliferating C2C12 cells overnight in the presence of 8 μg/mL of polybrene. Two µg/mL puromycin was added to select infected cells two days after infection. After 7 days of selection, we confirmed the successful CRISPR/Cas9-mediated gene editing by T7 endonuclease 1 digestion assay at the respective locus targeted by gRNA1 and 2. Cells were then cultured to confluence for myoblast differentiation.

### 4.3. Quantitative Real-Time PCR (qRT-PCR)

Total RNA was extracted from cells using TRIzol reagent. For qRT-RCR analysis, 1 μg of total RNA was reverse-transcribed to cDNA using InvitrogenTM M-MLV Reverse Transcriptase (28025013). Gene expression was assessed using iTaqTM Universal SYBR Green Supermix (Bio-Rad 1725125). Analysis was performed on Agilent Technologies Stratagene Mx3005P real-time PCR system with the following murine gene-specific primers listed in Table 1:

### 4.4. Cellular Protein Extraction, Subcellular Fractionation, and Western Blot

Cells were lysed in an RIPA buffer with a protease inhibitor and phosphatase inhibitor cocktail (Sigma Aldrich, St. Louis, MO, USA). Whole cell lysates were then centrifuged at 13,000 rpm for 15 min, and the pellet was discarded. Cytosol/nucleus fractionation was prepared as previously described [44]. Briefly, cells were washed with PBS and collected in ice-cold nuclei lysis buffer (10 mM Tris, 10 mM NaCl, 3 mM MgCl2, 0.5% NP-40, pH 7.6) with protease inhibitor cocktail. Lysates were then centrifuged at 600× *g* for 5 min. The supernatant was stored on ice (the cytosolic fraction). The pellet was washed once with ice-cold nuclei lysis buffer then resuspended in RIPA buffer with protease inhibitors. Lysates were centrifuged for 10 min at 16,000× *g*. The resulting supernatant was the nuclear fraction lysate. Protein concentration was determined by a Pierce BCA protein assay kit, and proteins were resolved by SDS-PAGE and transferred to a polyvinylidene fluoride (PVDF) membrane. After blocking with 5% fat-free milk for 1 h at room temperature, the membranes were incubated with the following primary antibodies diluted in 2.5% milk overnight at 4 °C: NAE1 (CST, #14321), UBC12 (CST, #5641), NEDD8 (CST, #2754), CUL1 (Scbt, sc-11384), pan-sarcomeric MyHC (i.e., Myosin (Skeletal, Fast), Sigma, clone MY-32, M4276)), MYOG (DSHB, F5D), ACTA1 (Proteintech, 17521-1-AP), ACTB (thermofisher, MAB1501), alpha-tubulin (Proteintech, 66031-1-lg), MYOD1 (Proteintech, 18943-1-AP), HDAC4 (Scbt, sc-46672), HDAC5 (Scbt, sc-133106), SIRT1 (CST, #8469). The corresponding HRP-conjugated secondary antibodies were applied for 1 h at room temperature. Then, the membranes were developed using ECL substrate and visualized using the GE A600 Imaging System followed by densitometry qualification using ImageQuantTL (GE Healthcare).

### 4.5. Immunofluorescence Microscopy

For immunofluorescence staining of cultured cells, cells were fixed in 4% paraformaldehyde (PFA) in PBS for 10 min, permeabilized with 0.2% Triton X-100 in PBS, then blocked with 3% BSA/PBS for 1 h at room temperature. Cells were incubated with primary antibodies against MyHC (Myosin (skeletal, fast), 1:500, Sigma, M4276 clone MY-32), NEDD8 (1:300, CST, #2754), SIRT1 (1:100, CST, #8469) overnight at 4 °C, followed by 1 h incubation with Alexa Fluor conjugated secondary antibody at room temperature in the dark. Nuclear counterstaining was performed with DAPI or Hoechst 33342. Microscopy was performed using a Keyence BZ-X800 fluorescence microscope.

### 4.6. Statistical Analysis

Data were shown as mean ± s.e.m. Statistical analysis was carried out using GraphPad Prism 8 software (GraphPad Software), including two-tailed unpaired Student’s *t*-test, one-way analysis of variance (ANOVA), or two-way ANOVA followed by a post-hoc test when appropriate. A *p*-value < 0.05 was considered statistically significant.

## Figures and Tables

**Table 1:** List of forward and backward primers used in this study.

	Forward Primers	Backward Primers
*Hdac4*	CAGGAGATGCTGGCCATGAA	GCACTCTCTTTGCCCTTCTC
*Hdac5*	GCTTCTTTGGACCAGAGTTCC	CATCTCAGTGGGGATGTTGG
*Sirt1*	CTTCAGTGTCATGGTTCCTT	ACCGAGGAACTACCTGATTA
*Myog*	GTCCCAACCCAGGAGATCATT	AGTTGGGCATGGTTTCGTCT
*36B4*	CGCTTTCTGGAGGGTGTCCGC	TGCCAGGACGCGCTTGTACC

The 2^−ΔΔCt^ method was used to analyze the relative fold changes in gene expression level normalized against 36B4 expression.

## Data Availability

The data presented in this study are available within the article text and figures.

## References

[1] Abidi N., Xirodimas D.P. (2015). Regulation of cancer-related pathways by protein NEDDylation and strategies for the use of NEDD8 inhibitors in the clinic. Endocr.-Relat. Cancer.

[2] Enchev R.I., Schulman B.A., Peter M. (2015). Protein neddylation: Beyond cullin-RING ligases. Nat. Rev. Mol. Cell Biol..

[3] Kandala S., Kim I.M., Su H. (2014). Neddylation and deneddylation in cardiac biology. Am. J. Cardiovasc. Dis..

[4] Gan-Erdene T., Nagamalleswari K., Yin L., Wu K., Pan Z.Q., Wilkinson K.D. (2003). Identification and characterization of DEN1, a deneddylase of the ULP family. J. Biol. Chem..

[5] Menon S., Chi H., Zhang H., Deng X.W., Flavell R.A., Wei N. (2007). COP9 signalosome subunit 8 is essential for peripheral T cell homeostasis and antigen receptor-induced entry into the cell cycle from quiescence. Nat. Immunol..

[6] Soucy T.A., Smith P.G., Milhollen M.A., Berger A.J., Gavin J.M., Adhikari S., Brownell J.E., Burke K.E., Cardin D.P., Critchley S. (2009). An inhibitor of NEDD8-activating enzyme as a new approach to treat cancer. Nature.

[7] Tateishi K., Omata M., Tanaka K., Chiba T. (2001). The NEDD8 system is essential for cell cycle progression and morphogenetic pathway in mice. J. Cell Biol..

[8] Park H.S., Ju U.I., Park J.W., Song J.Y., Shin D.H., Lee K.H., Jeong L.S., Yu J., Lee H.W., Cho J.Y. (2016). PPARgamma neddylation essential for adipogenesis is a potential target for treating obesity. Cell Death Differ..

[9] Su H., Li J., Zhang H., Ma W., Wei N., Liu J., Wang X. (2015). COP9 signalosome controls the degradation of cytosolic misfolded proteins and protects against cardiac proteotoxicity. Circ. Res..

[10] Zou J., Ma W., Li J., Littlejohn R., Zhou H., Kim I.M., Fulton D.J.R., Chen W., Weintraub N.L., Zhou J. (2018). Neddylation mediates ventricular chamber maturation through repression of Hippo signaling. Proc. Natl. Acad. Sci. USA.

[11] Vogl A.M., Brockmann M.M., Giusti S.A., Maccarrone G., Vercelli C.A., Bauder C.A., Richter J.S., Roselli F., Hafner A.S., Dedic N. (2015). Neddylation inhibition impairs spine development, destabilizes synapses and deteriorates cognition. Nat. Neurosci..

[12] Li L., Cao Y., Wu H., Ye X., Zhu Z., Xing G., Shen C., Barik A., Zhang B., Xie X. (2016). Enzymatic Activity of the Scaffold Protein Rapsyn for Synapse Formation. Neuron.

[13] Bhatia S., Pavlick A.C., Boasberg P., Thompson J.A., Mulligan G., Pickard M.D., Faessel H., Dezube B.J., Hamid O. (2016). A phase I study of the investigational NEDD8-activating enzyme inhibitor pevonedistat (TAK-924/MLN4924) in patients with metastatic melanoma. Investig. New Drugs.

[14] Shah J.J., Jakubowiak A.J., O’Connor O.A., Orlowski R.Z., Harvey R.D., Smith M.R., Lebovic D., Diefenbach C., Kelly K., Hua Z. (2016). Phase I Study of the Novel Investigational NEDD8-Activating Enzyme Inhibitor Pevonedistat (MLN4924) in Patients with Relapsed/Refractory Multiple Myeloma or Lymphoma. Clin. Cancer Res..

[15] Blondelle J., Shapiro P., Domenighetti A.A., Lange S. (2017). Cullin E3 Ligase Activity Is Required for Myoblast Differentiation. J. Mol. Biol..

[16] Lu J., McKinsey T.A., Zhang C.L., Olson E.N. (2000). Regulation of skeletal myogenesis by association of the MEF2 transcription factor with class II histone deacetylases. Mol. Cell.

[17] Sincennes M.C., Brun C.E., Rudnicki M.A. (2016). Concise Review: Epigenetic Regulation of Myogenesis in Health and Disease. Stem Cells Transl. Med..

[18] Liu N., Nelson B.R., Bezprozvannaya S., Shelton J.M., Richardson J.A., Bassel-Duby R., Olson E.N. (2014). Requirement of MEF2A, C, and D for skeletal muscle regeneration. Proc. Natl. Acad. Sci. USA.

[19] Bentzinger C.F., Wang Y.X., Rudnicki M.A. (2012). Building muscle: Molecular regulation of myogenesis. Cold Spring Harb. Perspect. Biol..

[20] Verdone L., Caserta M., Di Mauro E. (2005). Role of histone acetylation in the control of gene expression. Biochem. Cell Biol..

[21] Mathias R.A., Guise A.J., Cristea I.M. (2015). Post-translational modifications regulate class IIa histone deacetylase (HDAC) function in health and disease. Mol. Cell. Proteom..

[22] Mal A., Sturniolo M., Schiltz R.L., Ghosh M.K., Harter M.L. (2001). A role for histone deacetylase HDAC1 in modulating the transcriptional activity of MyoD: Inhibition of the myogenic program. EMBO J..

[23] Puri P.L., Iezzi S., Stiegler P., Chen T.T., Schiltz R.L., Muscat G.E., Giordano A., Kedes L., Wang J.Y., Sartorelli V. (2001). Class I histone deacetylases sequentially interact with MyoD and pRb during skeletal myogenesis. Mol. Cell.

[24] Lu J., McKinsey T.A., Nicol R.L., Olson E.N. (2000). Signal-dependent activation of the MEF2 transcription factor by dissociation from histone deacetylases. Proc. Natl. Acad. Sci. USA.

[25] Fulco M., Schiltz R.L., Iezzi S., King M.T., Zhao P., Kashiwaya Y., Hoffman E., Veech R.L., Sartorelli V. (2003). Sir2 regulates skeletal muscle differentiation as a potential sensor of the redox state. Mol. Cell.

[26] Fulco M., Cen Y., Zhao P., Hoffman E.P., McBurney M.W., Sauve A.A., Sartorelli V. (2008). Glucose restriction inhibits skeletal myoblast differentiation by activating SIRT1 through AMPK-mediated regulation of Nampt. Dev. Cell.

[27] Tanno M., Sakamoto J., Miura T., Shimamoto K., Horio Y. (2007). Nucleocytoplasmic shuttling of the NAD+-dependent histone deacetylase SIRT1. J. Biol. Chem..

[28] Miska E.A., Langley E., Wolf D., Karlsson C., Pines J., Kouzarides T. (2001). Differential localization of HDAC4 orchestrates muscle differentiation. Nucleic Acids Res..

[29] Pardo P.S., Boriek A.M. (2011). The physiological roles of Sirt1 in skeletal muscle. Aging (Albany NY).

[30] Rudnicki M.A., Schnegelsberg P.N., Stead R.H., Braun T., Arnold H.H., Jaenisch R. (1993). MyoD or Myf-5 is required for the formation of skeletal muscle. Cell.

[31] Hasty P., Bradley A., Morris J.H., Edmondson D.G., Venuti J.M., Olson E.N., Klein W.H. (1993). Muscle deficiency and neonatal death in mice with a targeted mutation in the myogenin gene. Nature.

[32] Nabeshima Y., Hanaoka K., Hayasaka M., Esumi E., Li S., Nonaka I., Nabeshima Y. (1993). Myogenin gene disruption results in perinatal lethality because of severe muscle defect. Nature.

[33] Shiraishi S., Zhou C., Aoki T., Sato N., Chiba T., Tanaka K., Yoshida S., Nabeshima Y., Nabeshima Y., Tamura T.A. (2007). TBP-interacting protein 120B (TIP120B)/cullin-associated and neddylation-dissociated 2 (CAND2) inhibits SCF-dependent ubiquitination of myogenin and accelerates myogenic differentiation. J. Biol. Chem..

[34] Mal A., Harter M.L. (2003). MyoD is functionally linked to the silencing of a muscle-specific regulatory gene prior to skeletal myogenesis. Proc. Natl. Acad. Sci. USA.

[35] Ohkawa Y., Marfella C.G., Imbalzano A.N. (2006). Skeletal muscle specification by myogenin and Mef2D via the SWI/SNF ATPase Brg1. EMBO J..

[36] Giacinti C., Bagella L., Puri P.L., Giordano A., Simone C. (2006). MyoD recruits the cdk9/cyclin T2 complex on myogenic-genes regulatory regions. J. Cell. Physiol..

[37] Kwon D.H., Eom G.H., Ko J.H., Shin S., Joung H., Choe N., Nam Y.S., Min H.K., Kook T., Yoon S. (2016). MDM2 E3 ligase-mediated ubiquitination and degradation of HDAC1 in vascular calcification. Nat. Commun..

[38] Lai Q.Y., He Y.Z., Peng X.W., Zhou X., Liang D., Wang L. (2019). Histone deacetylase 1 induced by neddylation inhibition contributes to drug resistance in acute myelogenous leukemia. Cell Commun. Signal..

[39] Pandey D., Hori D., Kim J.H., Bergman Y., Berkowitz D.E., Romer L.H. (2015). NEDDylation promotes endothelial dysfunction: A role for HDAC2. J. Mol. Cell. Cardiol..

[40] Potthoff M.J., Wu H., Arnold M.A., Shelton J.M., Backs J., McAnally J., Richardson J.A., Bassel-Duby R., Olson E.N. (2007). Histone deacetylase degradation and MEF2 activation promote the formation of slow-twitch myofibers. J. Clin. Investig..

[41] Nomura Y., Nakano M., Woo Sung H., Han M., Pandey D. (2021). Inhibition of HDAC6 Activity Protects against Endothelial Dysfunction and Atherogenesis in vivo: A Role for HDAC6 Neddylation. Front. Physiol..

[42] Peng L., Yuan Z., Li Y., Ling H., Izumi V., Fang B., Fukasawa K., Koomen J., Chen J., Seto E. (2015). Ubiquitinated sirtuin 1 (SIRT1) function is modulated during DNA damage-induced cell death and survival. J. Biol. Chem..

[43] Yu S., Xie L., Liu Z., Li C., Liang Y. (2019). MLN4924 Exerts a Neuroprotective Effect against Oxidative Stress via Sirt1 in Spinal Cord Ischemia-Reperfusion Injury. Oxid. Med. Cell. Longev..

[44] Luo L., Martin S.C., Parkington J., Cadena S.M., Zhu J., Ibebunjo C., Summermatter S., Londraville N., Patora-Komisarska K., Widler L. (2019). HDAC4 Controls Muscle Homeostasis through Deacetylation of Myosin Heavy Chain, PGC-1alpha, and Hsc70. Cell Rep..

